# Retraction of: Long noncoding RNA NR2F1-AS1 promotes the malignancy of non-small cell lung cancer via sponging microRNA-493-5p and thereby increasing ITGB1 expression

**DOI:** 10.18632/aging.206050

**Published:** 2024-07-31

**Authors:** Chan Zhang, Shangjie Wu, Rong Song, Changming Liu

**Affiliations:** 1 Department of Respiratory Medicine, The Second Xiangya Hospital of Central South University, Changsha 410000, Hunan, China; 2 Department of Respiratory Medicine, The Fourth Hospital of Changsha, Changsha 410006, Hunan, China; 3 Department of Anesthesiology, The Second Xiangya Hospital of Central South University, Changsha 410000, Hunan, China; 4 Department of Infectious Diseases, The First Hospital of Changsha, Changsha 410000, Hunan, China

**Keywords:** NR2F1 antisense RNA 1, microRNA-493-5p, ceRNA, non-small cell lung cancer

**This article has been retracted:** Aging has completed its investigation of this paper. Multiple concerns were raised about this paper, including overlap with unrelated papers from different institutions. We found overlap between the transwell images in Figures 5C and 6E and images from an earlier published unrelated paper [1]. We also found that the tumor images in Figure 7B were previously published in an unrelated paper from a different lab [2]. In addition, the same distinctively scratched ruler was used to measure xenograft tumors in a number of other papers from unrelated teams of authors [2, 3, 4]. The formats of the selected papers [3, 4] are nearly identical, with some figures being almost indistinguishable. Aging Scientific Integrity office contacted the authors but the authors did not respond to requests to clarify and the paper was retracted based on Editorial decision. The Scientific Integrity office also notified authors’ Institutions about this retraction and added their names to the Editorial Warning list.
